# Proteolytically Resistant Bioactive Peptide-Grafted Sr/Mg-Doped Hardystonite Foams: Comparison of Two Covalent Functionalization Strategies

**DOI:** 10.3390/biomimetics8020185

**Published:** 2023-04-29

**Authors:** Annj Zamuner, Elena Zeni, Hamada Elsayed, Michele Di Foggia, Paola Taddei, Antonella Pasquato, Lucy Di Silvio, Enrico Bernardo, Paola Brun, Monica Dettin

**Affiliations:** 1 Department of Civil, Environmental and Architectural Engineering, University of Padua, Via Marzolo 9, 35131 Padova, Italy; 2 Department of Industrial Engineering, University of Padua, Via Marzolo 9, 35131 Padova, Italymonica.dettin@unipd.it (M.D.); 3 Department of Biomedical and Neuromotor Sciences, University of Bologna, Via Irnerio 48, 40126 Bologna, Italy; 4 Faculty of Dentistry, Oral & Craniofacial Sciences King’s College London, London SE1 9RT, UK; 5 Department of Molecular Medicine, University of Padua, Via Gabelli 63, 35121 Padova, Italy

**Keywords:** bone tissue engineering, bioceramics, hardystonite, retro-inverted peptide, covalent grafting, osteoblasts

## Abstract

Hardystonite-based (HT) bioceramic foams were easily obtained via thermal treatment of silicone resins and reactive oxide fillers in air. By using a commercial silicone, incorporating strontium oxide and magnesium oxide precursors (as well as CaO and ZnO), and treating it at 1100 °C, a complex solid solution (Ca_1.4_Sr_0.6_Zn_0.85_Mg_0.15_Si_2_O_7_) that has superior biocompatibility and bioactivity properties compared to pure hardystonite (Ca_2_ZnSi_2_O_7_) can be obtained. Proteolytic-resistant adhesive peptide mapped on vitronectin (D2HVP), was selectively grafted to Sr/Mg-doped HT foams using two different strategies. Unfortunately, the first method (via protected peptide) was unsuitable for acid-sensitive materials such as Sr/Mg-doped HT, resulting in the release of cytotoxic levels of Zinc over time, with consequent negative cellular response. To overcome this unexpected result, a novel functionalization strategy requiring aqueous solution and mild conditions was designed. Sr/Mg-doped HT functionalized with this second strategy (via aldehyde peptide) showed a dramatic increase in human osteoblast proliferation at 6 days compared to only silanized or non-functionalized samples. Furthermore, we demonstrated that the functionalization treatment does not induce any cytotoxicity. Functionalized foams enhanced mRNA-specific transcript levels coding *IBSP*, *VTN*, *RUNX2*, and *SPP1* at 2 days post-seeding. In conclusion, the second functionalization strategy proved to be appropriate for this specific biomaterial and was effective at enhancing the material’s bioactivity.

## 1. Introduction

With the ageing population expected to rise to 2 billion by 2050 (United Nations World Population Ageing, 2017), the occurrence of degenerative diseases, such as osteoporosis, Alzheimer’s and cardiovascular disease, is increasing. Therefore, replacing diseased tissue to improve quality of life is paramount. Although the implementation of synthetic biomaterials, artificial devices, and organ transplantation has been able to meet demands so far, it has led to a large cost burden on health services, and concerns surrounding immunogenicity, biocompatibility, and organ shortage are inevitable [1]. Tissue engineering (TE) represents a potential solution, with the emergence of bone regeneration for orthopaedic conditions, skin reconstruction for burns, and drug delivery, to name a few. In particular, an engineered substitute for bone TE (BTE) requires a three-dimensional scaffold that provides the milieu to support and guide cell adhesion, growth, and proliferation in order to promote new tissue formation [2]. General criteria for designing a suitable scaffold graft for BTE include the material type, its porosity and architecture, and surface chemistry combined with its mechanical strength [3]. New approaches to BTE scaffold manufacturing are now emerging to boost scaffold performance and develop even more sustainable processing routes [4]. In this regard, a novel method based on the use of preceramic polymers containing micro- and nano-sized fillers has been developed to successfully produce highly porous bioactive glass scaffolds [5]. Highly amorphous bioceramic foams can be obtained from the thermal treatment of preceramic polymers, in the form of silicone resins, containing micro and nano-sized filler powders [5,6,7]. Simply by changing the starting nano-sized active fillers, this method enables one to obtain different types of ceramics such as wollastonite, diopside, akermanite, hardystonite, fluorapatite, etc. [7,8,9,10].

Recently, much interest has been devoted to developing multifunctional bioceramics that match the properties of porous bioactive scaffolds and the therapeutic value of ion release [4]. For this reason, bioactive glass has been doped with different trace elements to provide a smart strategy for in situ delivery with a controlled release kinetic. In fact, studies have demonstrated that the controlled release into the cellular ambiance of Sr, Cu, Mg, Co, or Ga could lead to therapeutic outcomes such as promoting angiogenesis or antibacterial effects [11,12,13]. Single-phase Sr/Mg-doped hardystonite (HT) bioceramic foams were successfully attained via the heat treatment of a silicone resin blended with reactive oxide fillers in air, and, according to preliminary biological assays, they demonstrated increased osteoblast biocompatibility and bioactivity with respect to pure HT [14].

Osseointegration and the long-term survival of a TE graft are driven by several reactions occurring at the tissue-scaffold interface. In BTE, one of the most critical factors is to produce an implant with similar properties to autogenous grafts in order to establish communication with the surrounding bioenvironment [15]. Research from the past decade indicates that the properties of the insoluble microenvironment can drive cell behaviors such as adhesion, morphology, proliferation, and differentiation. [16,17]. The ever-increasing knowledge concerning the molecular processes that drive cell adhesion and growth has focused on improving the surface–cell interactions of biomaterials according to the novel approach of “biochemical functionalization” [15]. Several techniques have recently been employed to maximize the biological performances of bone implants [18,19].

A very promising approach for bone substitutes involves decorating the scaffold surface with osteogenic growth factors or adhesive peptides [20] via sophisticated approaches to guarantee selective bonds and an adequate spatial orientation [21,22,23,24,25,26]. Peptides can be grafted to several surfaces (e.g., synthetic polymers, quartz, metal oxides, and glass), using specific strategies considering the type of chemically reactive groups available on the implant surface [27,28]. Furthermore, additional spacers between the implant surface and the peptide were introduced in order to confer to the latter higher flexibility for its interaction with cell receptors [29].

Several peptide sequences have been identified and used to enrich biomaterials to increase the biological properties of the latter. One of the most referenced among them is the tripeptide RGD, a cell-binding region in fibronectin that targets the integrin receptor and is able to induce the adhesion of different cell types [30,31]. Unlike RGD, a nonapeptide (named HVP) from the human Vitronectin protein (sequence 352–360) is able to selectively enhance osteoblast adhesion using an osteoblast-specific mechanism involving the interactions between membrane glycosaminoglycans (GAGs) and the heparin-binding sites on the proteins of the extracellular matrix (ECM) [32]. A retro-inverted dimeric analog (D2HVP) was synthesized with the aim of increasing the ionic interactions with cellular GAGs but while also avoiding the proteolytic degradation that normally occurs under physiological conditions [15]. In this study, the retro-inverted dimeric analogue of HVP was selectively grafted to Sr/Mg-doped HT, following two different chemical strategies. The first strategy (via a side-chain protected peptide) was based on an established protocol that showed promising results with wollastonite/diopside bioceramic foams [6], whilst the second strategy (via aldehyde peptide) was a novel strategy designed *ad hoc*, to avoid the use of organic solvent and acid treatments.

## 2. Materials and Methods

### 2.1. Materials

H62C, a preceramic precursor polymer, was obtained from Wacker-Chemie GmbH (Munich, Germany). SrCO_3_, CaCO_3_, and Mg(OH)_2_ were obtained from Industrie Bitossi (Vinci, Italy). Isopropanol (IPA), ZnO, N’-Dicarbamoylhydrazine (HZ), acetic acid (AcOH), methanol (MeOH), (3-Aminopropyl)triethoxysilane (APTES), sodium cyanoborohydride (NaCNBH_3_), Sieber Amide, and H-Phe-H NovaSyn^®^ TG resins, together with all Fmoc-protected D-amino-acids, ascorbic acid, dexametasone, β-glycerophosphate, bovine serum albumumin (BSA), MTT (3-(4,5-dimethylthia-zole-2-yl)-2,5-diphenyl tetrazoliumbromide, and a Lactate Dehydrogenase Activity Assay Kit were purchased from Merck KGaA (Darmstadt, Germany). 2-(1H-Benzotriazole-1-yl)-1,1,3,3-tetramethyluronium hexafluorophosphate (HBTU) and 1-Hydroxybenzotriazole (HOBt) were obtained from Advanced Biotech (Seveso, Italy). N,N-diisopropylethylamine (DIPEA), piperidine, N,N-dimethylformamide (DMF), N-methyl-2-pyrrolidone (NMP), dichloromethane (DCM), 2,2,2-trifluoroacetic acid (TFA), and acetonitrile were obtained from Biosolve (Leenderweg, Valkenswaard, The Netherlands). Triethoxysilane (TES) and acetone were provided by Sigma-Aldrich (Steinheim, Germany). Dulbecco’s Modified Eagle Medium (DMEM), heat-inactivated fetal bovine serum (FBS), penicillin, streptomycin, and trypsin-EDTA were obtained from Gibco (Thermo Scientific, Waltham, MA, USA). Carboxyfluorescein diacetate succinimidyl ester (CFSE) was purchased from Molecular Probe (ThermoFisher Scientific, Waltham, MA, USA). An SV Total RNA Isolation System kit was obtained from Promega (Milan, Italy). An iTaq Universal SYBR Green One-Step Kit was provided by Bio-Rad (Hercules, CA, USA, Stati Uniti).

### 2.2. Sr/Mg-Doped HT Foams’ Synthesis and Characterization

**Figure 1:**
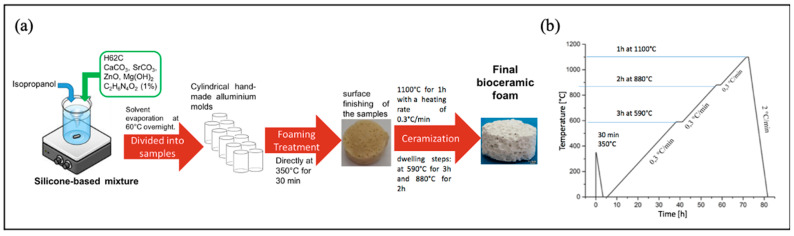
Bioceramic preparation. (**a**) Schematic representation of the Sr/Mg-doped HT foam synthesis and (**b**) thermal treatments.

Sr/Mg-doped HT foams were prepared using the following method. The recipe for 10 g of final HT consisted of 6.39 g of H62C (preceramic precursor polymer, a silica source, with a yield of 58%wt), 4.17 g of CaCO_3_ (<10 μm), 2.06 g of ZnO (<1.48 µm), 2.64 g of SrCO_3_ (<10 μm), and 1.76 g of Mg(OH)_2_ (<10 μm). All of the above chemicals were dissolved in 15 mL IPA. Next, we added 0.1 g (1% of the total final amount) of HZ (N′-Dicarbamoylhydrazine, or biurea; C_2_H_6_N_4_O_2_, 98%, foaming agent). The mixture was left at 60 °C overnight to dry all IPA. Following this, 1.8 g of the mixture was put in each melting pot of aluminum to proceed with two following treatments: (i) a foaming treatment at 350 °C for 30 min and (ii) a ceramization step with foams fired at 1100 °C for 1 h at a heating rate of 0.3 °C/min and dwelling steps at 590 °C for 3 h and 880 °C for 2 h. The cooling rate was set at 2 °C/min. A schematic representation of Sr/Mg-doped HT foam synthesis is reported in Figure 1.

The crystalline phase identification and both the physical and mechanical characterizations of Sr/Mg-doped HT have been reported elsewhere [14]. Bioceramic samples were cut into homogeneous rectangular shapes of 35 mg each before peptide grafting.

### 2.3. Peptides’ Synthesis and Characterization

#### 2.3.1. D2HVP

The D2HVP peptide (Sequence: H-Tyr-Gly-Lys-Arg-Asn-Arg-His-Arg-Phe-Tyr-Gly-Lys-Arg-Asn-Arg-His-Arg-Phe-NH_2_) was synthesized via standard Fmoc chemistry using Sieber Amide resin (0.72 mmol/g; scale 0.125 mmoles) and a fully automated peptide synthesizer (Syro I, Multisynthec, Witten, Germany). All amino acids were D-enantiomers. The side chain protections employed were Arg, Pbf; His and Asn, Trt; Lys, Boc; and Tyr, tBu. The coupling reaction was performed using 5 eq of Fmoc-protected D-amino acid, 5 eq of HOBt/HBTU, and 10 eq of DIPEA. All the couplings were double The peptide was cleaved from the solid support without contemporary side-chain deprotection using the following mixture: 1% TFA in DCM. A sample of the crude peptide was completely deprotected using the following mixture: 2.5% H_2_O MilliQ, 2.5% TES, and 95% TFA (90 min under magnetic stirring). After cleavage, the resin was filtered, the reaction mixture concentrated, and the crude peptide was precipitated with cold ethyl ether.

The chromatogram of the crude peptide was achieved under the following conditions: column, Vydac C_18_ monomeric (5 µm, 300 Å, 4.6 × 250 mm); injection volume, 20 µL of 1 mg/mL peptide solution; flow rate, 1 mL/min; eluent A, 0.05% TFA in water; eluent B, 0.05% TFA in CH_3_CN; and gradient, from 8% B to 18% B in 20 min, detection at 214 nm. The retention time was 12.4 min. The peptide identity was ascertained through Mass Spectrometry: experimental mass: 2448.32 Da and theoretical mass: 2447.82 Da (ESI-TOF, Mariner System 5220, Applied Biosystem, Perkin-Elmer, Foster City, CA, USA). The peptide used for bioceramic foam functionalization was the side-chain protected crude peptide.

#### 2.3.2. D2HVPF

The D2HVPF peptide with an aldehyde group at the C-terminus (Sequence: H-Tyr-Gly-Lys-Arg-Asn-Arg-His-Arg-Phe-Tyr-Gly-Lys-Arg-Asn-Arg-His-Arg-Phe-Phe-CHO) was synthesized, characterized, and purified as follows. The peptide was synthesized via standard Fmoc chemistry using H-Phe-H NovaSyn^®^ TG resin (0.18 mmol/g; scale 0.09 mmoles) and a fully automated peptide synthesizer (Syro I, Multisynthec, Witten, Germany). The side-chain protections employed were Arg, Pbf; His and Asn, Trt; Lys, Boc; and Tyr, tBu. The coupling reaction was carried out using 5 eq of Fmoc-protected D-amino acid, 5 eq of HOBt/HBTU, and 10 eq of DIPEA. All the couplings were single (45 min/each). The side-chain protections were removed using 5 mL of pure TFA for 1 h at room temperature under magnetic stirring. The peptide was subsequentially cleaved from the resin using 4 mL of the following mixture: AcOH/H_2_O MilliQ/DCM/MeOH (10:5:63:21) for 1 h at room temperature under magnetic agitation. Following this, the solution was filtered and collected to be lyophilized overnight. The obtained lyophilized peptide was then treated as follows: 2.5% H_2_O MilliQ, 2.5% TES, and 95% TFA (90 min, under magnetic stirring). The reaction mixture was concentrated, and the crude peptide was precipitated with cold ethyl ether. The peptide’s identity and homogeneity were ascertained using mass (4800 MALDI-TOF/TOF^TM^ Analyzer, AB Sciex Pte Ltd., Singapore, with 4000 Series Explorer^TM^ software, Applied Biosystems, Foster City, CA, USA) and reverse-phase high-performance liquid chromatography (RP-HPLC) analyses (Waters Corporation, Milford, MA), respectively. The chromatogram of the crude peptide was carried out under the following conditions: column, Vydac 238EV54 Everest (5 µm, 300 Å, 4.6 × 250 mm); injection volume, 100 µL of 1 mg/mL peptide solution; flow rate, 1 mL/min; eluent A, 0.05% TFA in water; eluent B, 0.05% TFA in CH_3_CN; and gradient, from 10% B to 25%B in 30 min, detection at 214 nm. The retention time results 17.6 min. Experimental mass: 2580.59 Da, Theoretical mass: 2579.97 Da.

The crude product was purified through RP-HPLC as follows. A total of 30 mg of crude peptide dissolved in 15 mL of Milli-Q water was loaded on Jupiter C18 (5 µm, 300 Å, 10 × 250 mm) and separated in the following conditions: Eluent A, 0.05% TFA in Milli-Q water; Eluent B, 0.05% TFA in CH_3_CN; gradient, from 0%B to 10%B in 2 min, then from 10%B to 25%B in 45 min; flow rate, 4 mL/min; and detection at 214 nm. The final homogeneity was >98%.

### 2.4. Bioceramic Functionalization and Characterization

#### 2.4.1. Functionalization with D2HVP via a Side-Chain Protected Peptide

**Figure 2:**
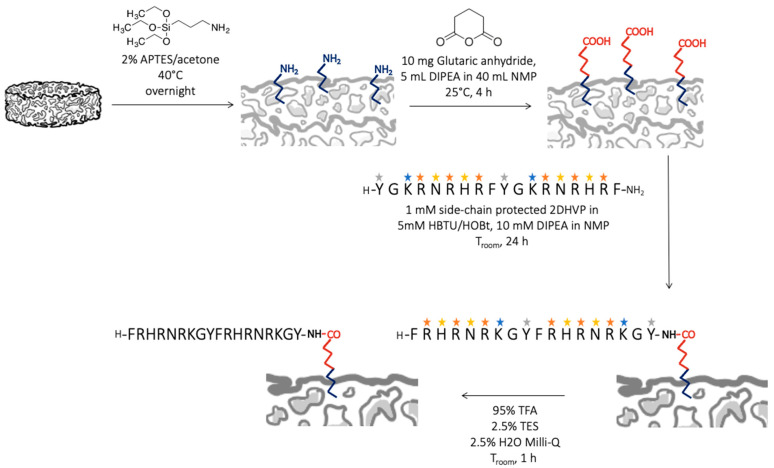
Scheme of the covalent and specific functionalization of Sr/Mg-doped HT foams with D2HVP via a side-chain protected peptide.

A specific functionalization of Sr/Mg-doped HT foams (35 mg) was obtained following the procedure reported in Figure 2. The last passage of the scheme in Figure 2 is the side-chain deprotection that was achieved by treating each sample with a solution of 2.5% MilliQ water and 2.5% TES in TFA for 1 h at room temperature. Eventually, the samples were washed once in TFA, twice in acetone, and three times in MilliQ water and finally twice in acetone. Foams were dried under vacuum for 30 min.

Previous studies [23] conducted on titanium surfaces proved that this functionalization strategy leads to a superficial peptide density between 0.016 and 0.063 nmol/cm^2^.

#### 2.4.2. Functionalization with D2HVP via Aldehyde Group

Sr/Mg-doped HT foams (35 mg each) were covalently and selectively functionalized with D2HVPF using the following protocol. Sr/Mg-doped HT foams were silanized overnight using 2% APTES in acetone solution at 40 °C. Consequently, silanized foams were treated overnight with 1 μM D2HVPF and 3 mg/mL of NaCNBH_3_ in 20 mM NaH_2_PO4·H_2_O and 200 mM NaCl buffer solution at pH 7.5 at room temperature.

D2HVPF was specifically synthesized carrying in the C-terminus an aldehyde group that enables a selective anchoring to the exposed amine groups on the silanized surface and does not require side-chain protections, as in [6].

The imines were then reduced to amines using NaCNBH_3_. Eventually, the functionalized foams were rinsed with Milli-Q water three times to remove all the unreacted reagents and were finally dried under vacuum.

**Figure 3:**
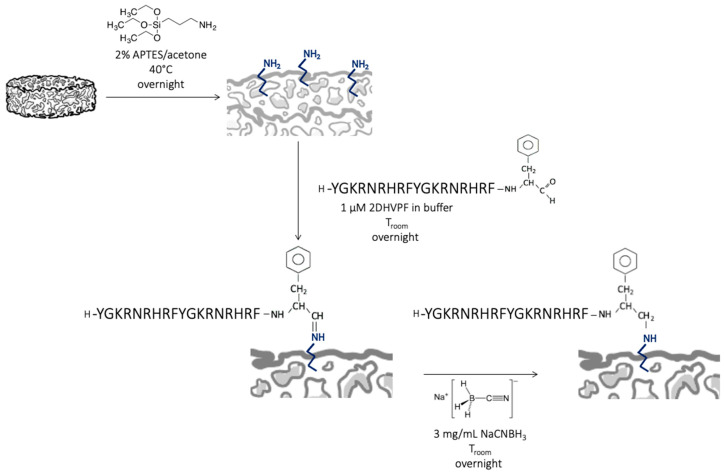
Covalent and selective functionalization. The aldehyde group at the D2HVPF C-terminal site reacted selectively with the aminic group on Sr/Mg-doped HT bioceramic silanized surface, forming an imine that was consequently reduced to an amine using NaCNBH_3_.

In contrast to the functionalization chemistry, requiring side-chain protected peptides reported in Section 2.4.1 (Figure 2), this procedure involves fewer steps and does not, therefore, necessitate the use of any organic solvent or acid treatment (Figure 3).

This functionalization strategy was shown to be effective in achieving a surface peptide density of 0.0859 × 10^−2^ nmol/cm^2^ on a silanized surface model [33].

### 2.5. Vibrational Raman and IR Spectroscopy

Vibrational Raman and IR spectroscopies was used to assess the efficacy of the novel anchoring strategy via aldehyde peptide. Raman spectra were registered on a Jasco NRS-2000C spectrometer equipped with a microscope with 100× magnification. The spectra were recorded under backscattering conditions with 4 cm^−1^ spectral resolution using the 532 nm line (DPSS laser driver, LGBlase LLC, Fremont, CA, USA) with a power of about 2 mW. The detector was a liquid nitrogen-cooled CCD (Spec-10:100B, Roper Scientific, Inc., Tucson, AZ, USA). Each spectrum was the average of three measurements recorded at three different points of each sample.

IR spectra were recorded in triplicate on a Shimadzu IRTracer-100 Fourier Transform FTIR spectrometer equipped with a QATR-10 single crystal diamond Attenuated Total Reflectance (ATR) accessory and a Deuterated Lanthanum α-Alanine doped TriGlycine Sulphate (DLaTGS) detector; the spectral resolution was 4 cm^−1^ with 64 scans for each spectrum.

### 2.6. Inductively Coupled Plasma Mass Spectroscopy (ICP-MS)

ICP analysis was conducted to evaluate the eventual ions released from functionalized HT samples. Silanized HT was used as control. Each sample (437.04 ± 9.38 mg) was immersed in 2.4 mL of 20 mM NaH_2_PO_4_·H_2_O and 200 mM NaCl buffer. The time points were 2 h, 18 h, 42 h, and 48 h. At each time point, 100 μL were selected for analysis, and the volume of buffer was restored to 2.4 mL. Inductively coupled plasma mass spectroscopy (ICP-MS, Agilent 7500 cx, Agilent Technologies, Santa Clara, CA, USA) was used to measure the concentration of the released ions.

### 2.7. Scanning Electron Microscopy (SEM)

Functionalized HT foams were left for 4 months in 20 mM NaH_2_PO4·H_2_O and 200 mM NaCl buffer and were then removed from the buffer and completely dried in air. Samples were sputter coated with gold (EMITECHK950x Turbo Evaporator, EBSciences, East Granby, CT, USA) and observed by means of Scanning Electron Microscopy (SEM; Cambridge Stereoscan 440 SEM, Cambridge, UK). Images were acquired at 30X and 500X magnifications with an accelerating voltage of 20 kV.

### 2.8. Biological Assays

#### 2.8.1. Cell Culture

Primary human (h) osteoblast cells were obtained from explants of cortical mandible bones collected during a surgical procedure from healthy subjects. The study was approved by the Ethical Committee of the University Hospital of Padova (research protocol No. 4899/AO/20 approved on 5 May 2020). Patients were informed of the study aims and protocol and provided their written informed consent. Bone fragments were cultured in DMEM supplemented with 20% heat-inactivated FBS, 10,000 units/mL of penicillin, and 10,000 µg/mL of streptomycin (complete media) and incubated at 37 °C until cells migrated from the bone fragments. At cell confluence, bone fragments were removed and cells were detached using trypsine-EDTA and cultured in DMEM supplemented with 50 mg/mL ascorbic acid, 10 nM dexamethasone, and 10 mM β-glycerophosphate. The osteoblast phenotype was confirmed via the von Kossa staining [34]. Cells were used in passages 3 and 8. For biological assays, bioceramic foams (35 mg each) were placed into non-treated sterile plates (Sarstedt, Nümbrecht, Germany) and incubated with h-osteoblasts (3 × 10^5^ cells/cm^2^) in the supplemented culture medium.

#### 2.8.2. Cell Staining

H-osteoblasts were stained with CellBrite Green to label cell cytoplasmic membranes with fluorescent green dye. Before cell seeding, CellBrite Green was added directly to the culture medium in the tissue culture flask in an adequate concentration (1:100) and incubated overnight.

#### 2.8.3. EDU Proliferation Assay

A total of 1 × 10^5^ h-osteoblasts suspended in osteoblast growth medium were seeded onto Sr/Mg-doped HT foams functionalized via protected peptide D2HVP on a 48-well plate. The plate was placed in a 37 °C 5% CO_2_ incubator for 24 h and was subsequently treated with the initial pulse of EDU (5-ethynyl-2′-deoxyuridine), according to the protocol outlined in Click-iT proliferation kit (ThermoFisher Scientific, Waltham, MA, USA). After 48 h in a 37 °C 5% CO_2_ incubator, cells were fixed. Fixation was carried out using a cross-linking agent, (paraformaldehyde, PFA). PFA (2%) in PBS enriched with Ca^2+^, and Mg^2+^ was then added and placed under agitation at room temperature for 15 min. The 2% PFA solution was then removed, and cells were washed with PBS enriched with Ca^2+^ and Mg^2+^ twice. The cells were then counterstained with Hoechst (Sigma-Aldrich, St. Louis, MO, USA, dilution 1:2000 in PBS plus Ca^2+^ and Mg^2+^ for 10 min) for nuclei staining.

Imaging was performed using the Operetta High-Content imaging system (PerkinElmer, Waltham, MA, USA). EDU is a thymidine nucleoside analogue, representing an index of cell proliferation, as cells grown in the presence of EDU incorporate a thymidine base during the S phase, which is attached to a fluorophore label and allowing detection of proliferation cumulatively after the addition of the initial EDU pulse. The experiment was repeated in triplicate.

#### 2.8.4. CFSE Proliferation Assay

To assess cell proliferation on Sr/Mg-doped HT functionalized via aldehyde peptide D2HVPF, h-osteoblasts were first loaded with 25µM Carboxyfluorescein succinimidyl ester (CFSE), a cell-permeable fluorescent probe that is equally partitioned among daughter cells. Cells were incubated with CFSE at 37 °C for 10 min in pre-warmed PBS containing 0.1% v/v BSA, and the reaction was stopped by adding 5 volumes of ice-cold culture media. Cells were then washed, counted using Trypan blue, and seeded on bioceramic foams. After 2, 4, and 6 days on culture, cells were detached using trypsine-EDTA. Cell proliferation was assessed using a BD FACS-Calibur flow cytometer by evaluating the percentage of CFSE-positive cells.

#### 2.8.5. Cytotoxicity Assay

To investigate whether functionalized Sr/Mg-doped HT foams were cytotoxic, we evaluated cell death by measuring lactate dehydrogenase (LDH), a soluble cytoplasmic enzyme released into extracellular space when membranes are damaged [35]. Osteoblasts were seeded on bioceramic foams for 4 or 6 days, and the conditioned media were harvested and combined with lysis buffer, following the instructions of the Lactate Dehydrogenase Activity Assay Kit (Merck, Darmstadt, Germany). Samples were incubated at 37 °C for 45 min and centrifuged to eliminate cell debris. Supernatants were incubated with LDH assay buffer, and the reaction was stopped 30 min later via acidification. Absorbance was measured at 520 nm using a plate reader (MultiPlateReader VictorX2, Perkin Elmer, CA, USA). Positive control was set by incubating cells for 30 min with 1% Triton, a membrane detergent agent.

#### 2.8.6. Quantitative Real Time Polymerase Chain Reaction

Specific mRNA transcript levels coding human Integrin Binding Sialoprotein (*IBSP*), human Vitronectin (*VTN*), Secreted Phosphoprotein 1 (*SPP1*), and Runt-Related Transcription Factor 2 (*RUNX2*) were quantified in osteoblast cells cultured for 48 h on bioceramic foams. At the end of incubation, total RNA was extracted using the SV Total RNA Isolation System kit. Contaminating DNA was removed via DNase I digestion. cDNA synthesis and subsequent polymerization were performed using the iTaq Universal SYBR Green One-Step Kit. The reaction mixture contained 200 nM forward primer, 200 nM reverse primer, iTaq universal SyBR Green reaction mix, iScript reverse transcriptase, and 200 ng total RNA. Real-time PCR was performed using ABI PRISM 7700 Sequence Detection System (Applied Biosystems, ThermoFisher Scientific, Waltham, MA, USA). Human GAPDH was used as a reference gene. Target and reference genes were amplified with efficiencies near 100%. Oligonucleotides used for PCR are listed in Table 1.

#### 2.8.7. Statistical Analysis

Data are expressed as mean ± standard deviation of at least three experiments. Statistical analysis was performed using the GraphPad Prism software (GraphPad Software Inc., La Jolla, CA, USA), and statistical significance was calculated using one-way analysis of variance (ANOVA) and Tukey’s multiple comparisons test. The level of statistical significance was set at *p*-value ≤ 0.05.

## 3. Results

### 3.1. Vibrational Raman and IR Spectroscopies

Vibrational Raman and IR spectroscopies showed that both the selective and covalent functionalization strategies were successful. In both cases, bands assignable to the D2HVP peptide were identified on the surface of Sr/Mg-doped HT using the vibrational techniques.

**Figure 4:**
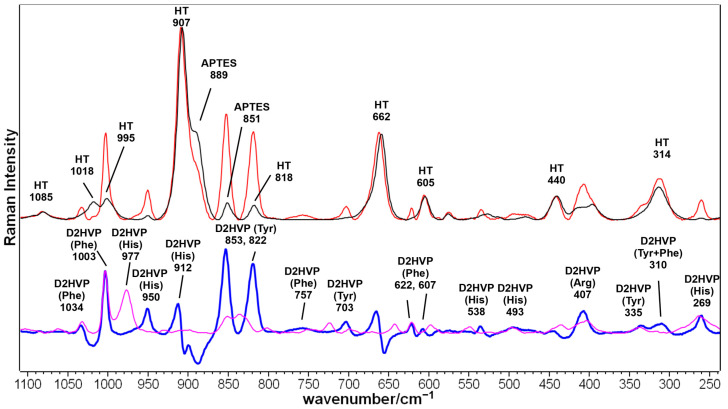
Raman spectra of the Sr/Mg-doped HT foams obtained after silanization treatment (black line) and after the covalent grafting via aldehyde peptide D2HVPF (red line). The difference between the functionalized and the silanized bioceramic spectra is reported below (blue line) and compared to the Raman spectrum of the D2HVP peptide alone (purple line). The bands assignable to the bioceramic substrate (HT) and to amino acid residues belonging to the D2HVP peptide (tyrosine (Tyr), histidine (His), phenylalanine (Phe), and arginine (Arg)) are indicated.

Figure 4 shows the Raman spectra of the Sr/Mg-doped HT foams obtained after silanization treatment and after the covalent grafting via aldehyde peptide D2HVPF. In the spectrum of the control substrate, the bands attributed to HT [36] and APTES [37] were assigned according to the literature. The spectrum of the functionalized sample shows the bands of the bioceramic substrate and D2HVP. The bands attributed to the peptide were better evidenced after spectrum subtraction (i.e., functionalized HT minus only silanized HT (blue spectrum in Figure 4)) and were compared with the Raman spectrum of the D2HVP peptide alone (purple spectrum in Figure 4) to confirm functionalization. Similar to the spectrum of the peptide alone, the difference spectrum was dominated by the bands of aromatic amino acids, i.e., tyrosine (853, 822, 703, 335, and 310 cm^−1^), histidine (950, 912, 538, 493, and 269 cm^−1^), and phenylalanine (1034, 1003, 757, 622, and 607 cm^−1^) as well as arginine (407 cm^−1^) [38]. The shift of some bands, such as those relative to histidine in the 980–910 cm^−1^ spectral region, can be attributed to the interaction with the surface (orientational effects) and hydrogen bonding [39,40]. Moreover, the Raman tyrosine doublet at about 850–830 cm^−1^ has been widely used to describe the average hydrogen-bonding state of the tyrosine phenoxyl groups (and, therefore, whether the tyrosine residues are buried or exposed) in globular proteins. The calculated I_853_/I_822_ intensity ratio was about 1.2, similar to the value previously reported for vitronectin adsorbed on nanophase alumina [41], indicating that the phenolic OH group acts as both a donor and an acceptor of moderate/weak strength H-bonds. The absence of the bands attributed to vibrations of the peptide bond (i.e., the Amide I in the 1700–1620 cm^−1^ range and Amide III in the 1300–1225 cm^−1^ region) has been previously observed for the functionalization of an Mn-containing bioactive glass with a BMP-2 mimicking peptide [42] and is therefore not surprising.

**Figure 5:**
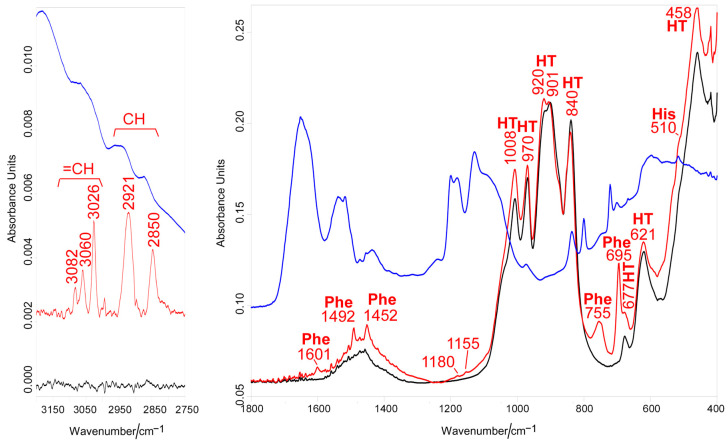
IR spectra of the Sr/Mg-doped HT foams obtained after silanization treatment (black line) and after the covalent grafting via aldehyde peptide D2HVPF (red line). The spectrum of the pure D2HVP peptide is reported for comparison (blue line). The bands assignable to the bioceramic substrate (HT) and to amino acid residues belonging to the D2HVP peptide (histidine (His) and phenylalanine (Phe)) are indicated.

IR spectroscopy confirmed the effective anchoring of the peptide to the Mg/Sr-doped HT substrate. Figure 5 shows the IR spectra of the Sr/Mg-doped HT foams obtained after silanization treatment and after the covalent grafting via aldehyde peptide D2HVPF. The spectrum of the pure D2HVP peptide is reported for comparison. The spectrum of the grafted foam displayed the bands of Sr/Mg-doped HT (assigned according to the literature [43]) together with those of aromatic amino acids, i.e., phenylalanine and histidine, belonging to the D2HVP peptide. A previous study has already observed the non-detection of the strong Amide I and II modes of the peptide (at about 1650 and 1540 cm^−1^) [42]. According to the literature [44], the weak spectral features at 1180 and 1155 cm^−1^ may be assigned to the C-N stretching mode of secondary amines and would confirm the reduction of the Schiff’s base, according to the reaction scheme reported in Figure 3.

The co-detection of bands assignable to the Sr/Mg-doped HT substrate as well as to D2HVP suggests that the thickness of the peptide layer was lower than 2 microns (i.e., the sampling depth of the diamond ATR technique).

Figures S1 and S2, Supplementary Material, show the Raman and IR spectra recorded on the surface of Sr/Mg-doped HT foams as obtained after silanization treatment and after the covalent grafting via side-chain protected peptide D2HVP. The trend of the spectra was highly influenced by the acidic treatment with trifluoroacetic acid used in this strategy.

Both the Raman and IR spectra of the functionalized sample show the bands of Sr/Mg-doped HT; strong spectral features assignable to the trifluoroacetate ion were detected both in Raman (at 1456, 853, 726, 601, 440, 414, and 267 cm^−1^ [45,46]) and IR spectra (at 1671, 1458, 1198–1139 and 727 cm^−1^ [46,47]). According to the literature [47], this result indicates that the amine groups of basic amino acids underwent protonation upon treatment with trifluoroacetic acid, and ionic interactions between protonated amine groups and trifluoroacetate formed.

In the Raman spectrum, the D2HVP anchoring was confirmed through the presence of the tyrosine doublet (853–822 cm^−1^) and histidine band at 960 cm^−1^ [38]. Due to the superposition of the trifluoroacetate band at about 850 cm^−1^, it was impossible to use the I_853_/I_822_ intensity ratio to study the H-bond status of tyrosine, as performed for the previous sample.

Other new bands appeared after grafting, i.e., around 490 cm^−1^ in Raman and at 3400, 1043, and 800 cm^−1^ in IR (with a strengthening of the 452 cm^−1^ component). They may be related to the partial degradation of the ceramic substrate (see discussion below) due to the acidic treatment; the above mentioned bands are compatible with the presence of a silica-based material [48,49,50], which is reported as the product of the acidic treatment of pyrosilicates [51].

### 3.2. Biological Assays

#### 3.2.1. Sr/Mg-Doped HT Foams Functionalized via a Side-Chain Protected Peptide Showed No Osteoblasts Proliferation

**Figure 6:**
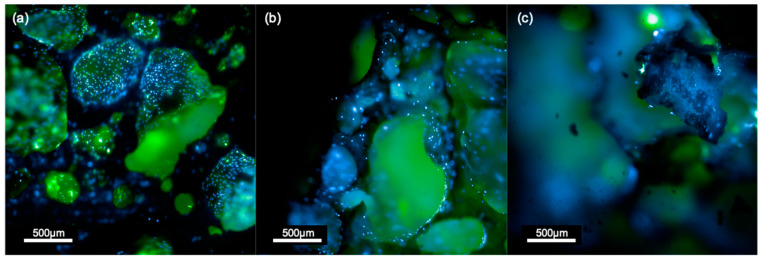
H-osteoblast on pristine Sr/Mg-doped HT (**a**), silanized Sr/Mg-doped HT (**b**), and Sr/Mg-doped HT foams functionalized via side-chain protected peptide D2HVP (**c**) at 6 days from the seeding. Blue is Hoechst (cell nuclei), and green is CellBrite Green (cytoplasmic membranes). Scale bar = 500 μm.

**Figure 7:**
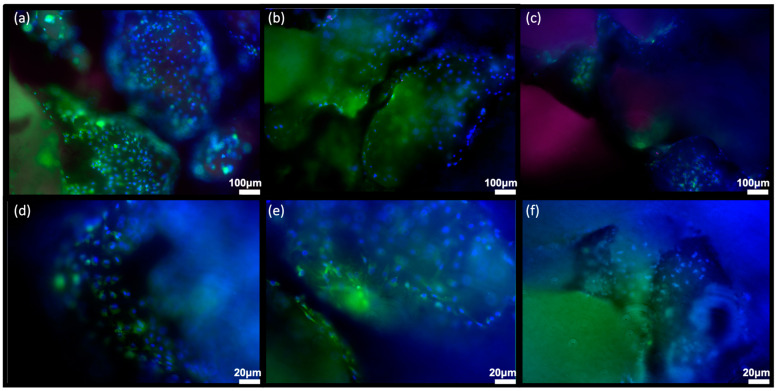
Human Osteoblast seeded on (**a**–**d**) Sr/Mg-doped hardystonite foams, (**b**–**e**) silanized Sr/Mg-doped hardystonite foams, and (**c**–**f**) D2HVP functionalized Sr/Mg-doped hardystonite foams. Images were taken in fluorescent microscopy with Olympus 1X51microscope (Evident Corporation, Shinjuku-ku, Tokyo, Japan) (**a**–**c**) 10× magnification, scale bar = 100 µm, and (**d**–**f**) 20× magnification, scale bar = 20 µm. Each image corresponds to a z-stack. Blue corresponds to the nuclei (Hoechst), Green to the entire cell (CellBrite Green), and Red nuclei correspond to EDU+ cells.

H-osteoblast seeded on Sr/Mg-doped HT foams functionalized via a side-chain protected peptide showed no proliferation at 6 days post-seeding (Figure 6c), and cells were not clearly visible on the bioceramic surface. Cells were visible and present in greater numbers on silanized scaffolds (Figure 6b and Figure 7b–e) and even more on the surface of pristine Sr/Mg-doped HT (Figure 6a and Figure 7a–d). The Sr/Mg-doped HT foams were completely colonized by h-osteoblast 6 days post-seeding (Figure 6a and Figure 7a–d), indicating that the bioceramic material formulation does not induce cytotoxicity.

This result is therefore explained by the ICP analysis, especially regarding the release of Zn^2+^, which, if de-adsorbed at concentrations higher than 0.050 mg/mL, can induce cytotoxicity.

#### 3.2.2. ICP-MS Analysis

**Figure 8:**
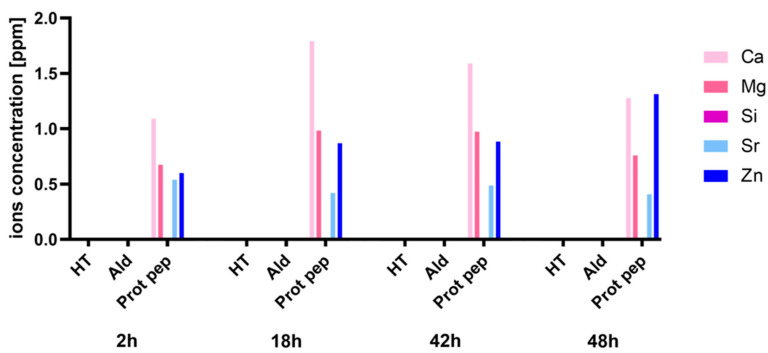
Ions released from functionalized Sr/Mg-doped HT foams. “HT” stands for silanized HT, “Ald” represents HT samples functionalized via aldehyde peptide D2HVPF, and “Prot pep” stands for HT samples functionalized via protected peptide D2HVP.

The ions released from functionalized Sr/Mg-doped HT in PBF solution (20 mM NaH_2_PO_4_·H_2_O and 200 mM NaCl) at different time points (2 h, 18 h, 42 h, and 48 h) is reported in Figure 8.

As clearly evident from Figure 8, only HT foams functionalized via protected peptide showed a consistent ion release at all time points. No ion release was detected for silanized HT and HT foams functionalized via aldehyde peptide.

**Figure 9:**
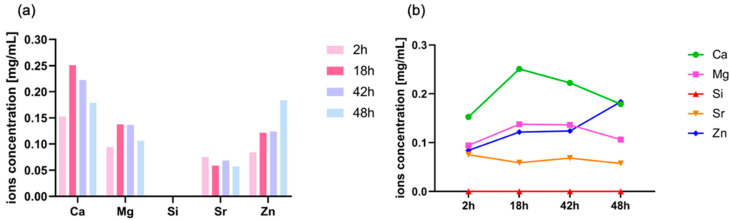
Ions released from HT samples functionalized via protected peptide D2HVP (**a**). Degradation profiles of HT samples functionalized via protected peptide D2HVP (**b**).

Looking at the concentration values of the released Zn^2+^ (Figure 9a,b), it is clear that they are always higher than the cytotoxic level of 0.05 mg/mL [52,53].

The concentration of silicon released from Sr/Mg-doped HT is very low. This is quite surprising since the material belongs to the family of sorosilicates, so that Ca^2+^ and Sr^2+^ are sandwiched between ((Zn, Mg)Si_2_O_7_)^2−^ layers. The release of Zn^2+^ and Mg^2+^ ions should lead to the dismantling of layers, with silicate ions in solution, as found for (not functionalized) akermanite (Ca_2_MgSi_2_O_7_) [54]. The interaction with the functional coating likely anchored silicate anions.

#### 3.2.3. SEM Analysis

**Figure 10:**
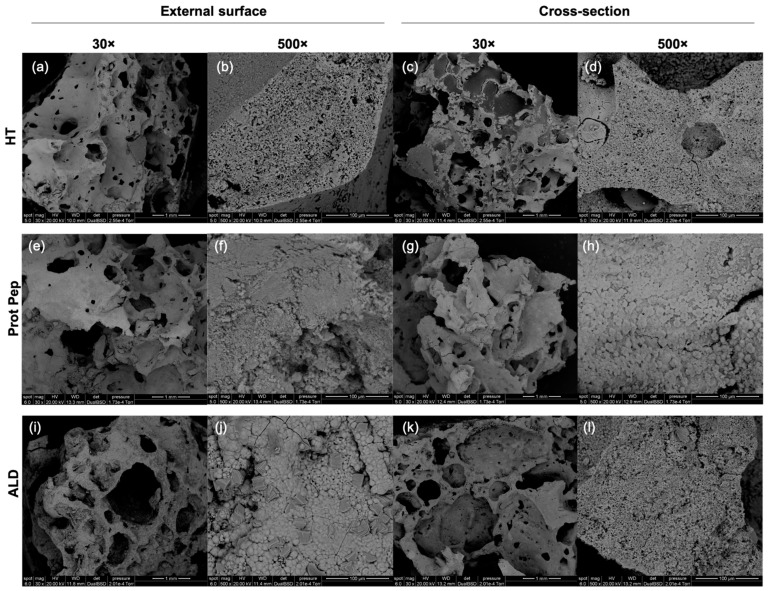
SEM images of silanized Sr/Mg-doped HT (HT) (**a**–**d**), Sr/Mg-doped HT functionalized via protected peptide D2HVP (Prot Pep) (**e**–**h**), and Sr/Mg-doped HT functionalized via aldehyde peptide D2HVPF (ALD) (**i**–**l**). The images were taken both on the external surface and on the cross-section of the foams at two different magnifications (30× and 500×). Scale bars are 1 mm (30× magnification) and 100 μm (500× magnification).

Images from SEM analysis of functionalized HT foams are reported in Figure 10.

From SEM results, it is evident that both functionalization treatments did not induce modifications in the macroscopic porous structure of the Sr/Mg-doped HT foams. Looking at the samples functionalized via protected peptide, at higher magnification (500×), the outer surface appears to be less compact and more crumbled than the other samples (HT and ALD).

EDS profiles confirmed the presence of the expected elements in all the samples (Figures S3–S5, Supplementary Materials).

#### 3.2.4. Functionalized Foams via Peptide-Aldehyde Enhanced Osteoblasts Proliferation without Inducing Cytotoxicity

**Figure 11:**
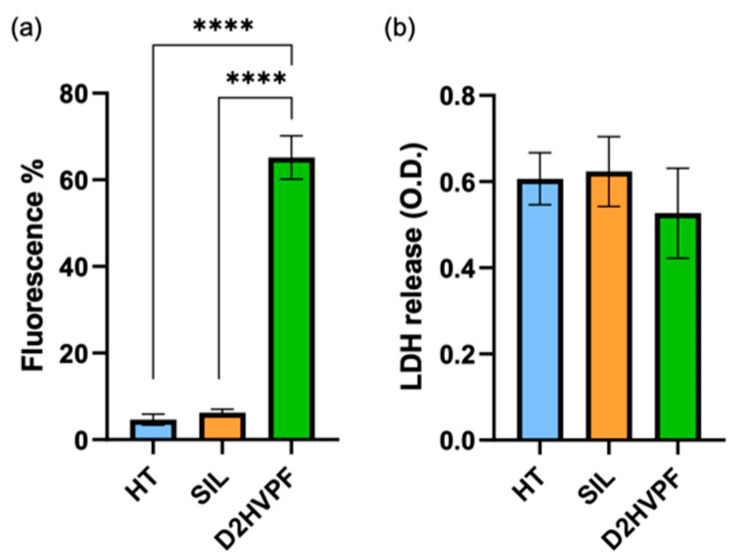
Proliferation and cytotoxicity assays. (**a**) H-osteoblast proliferation was assessed on functionalized Sr/Mg-doped HT foams, determined at 6 days in culture using the CFSE fluorescent probe. The percentage of fluorescent cells was determined via FACS analysis on 10,000 collected events. (**b**) LDH test at 6 days demonstrated the absence of cytotoxicity induced via functionalization treatment. HT refers to non-functionalized Sr/Mg doped HT foams, and SIL refers to silanized scaffolds. **** *p*-value < 0.0001.

Sr/Mg-doped HT foams functionalized with D2HVPF showed 14-fold increased cell proliferation with respect to the non-functionalized (HT) and silanized (SIL) foams (Figure 11a).

LDH assay at 6 days post-seeding confirmed the complete absence of any cytotoxic effect induced by the functionalization treatment (Figure 11b).

#### 3.2.5. Functionalized Foams Induce Gene Expression of Human Osteoblasts

**Figure 12:**
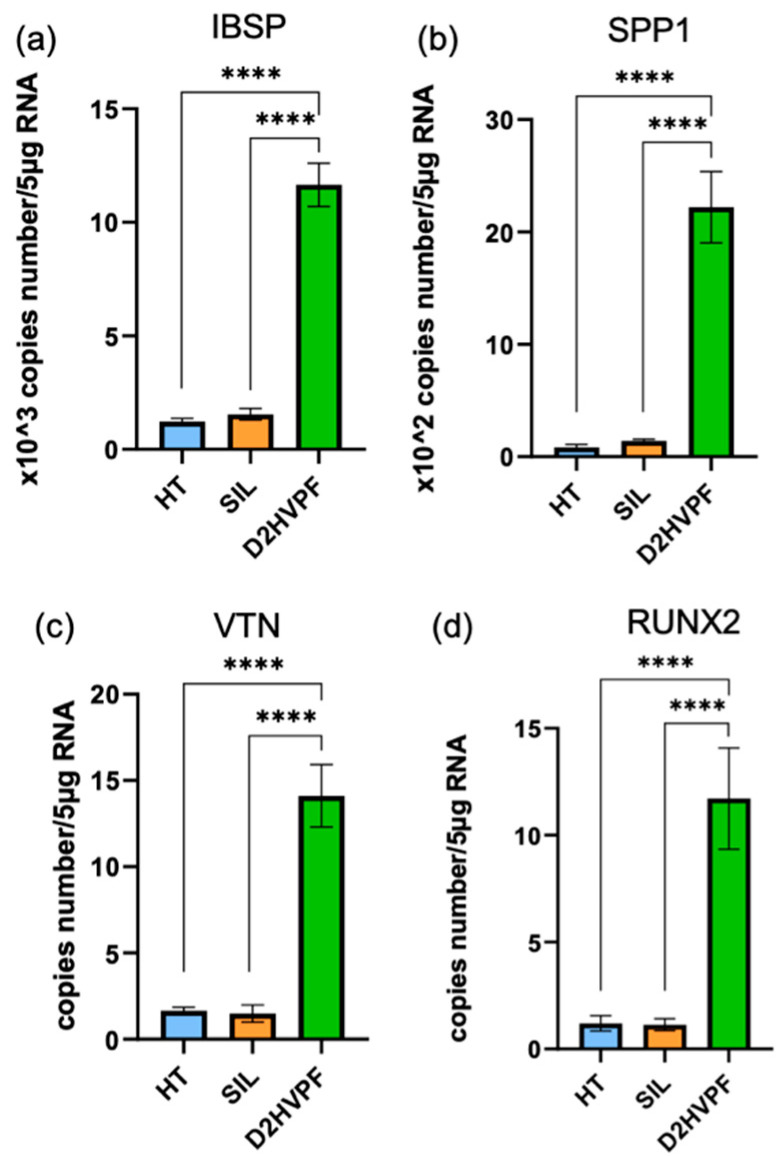
Gene expression. (**a**) Human *IBSP* (Integrin-Binding Sialoprotein), (**b**) Human *SPP1* (secreted phosphoprotein 1, Osteopontin), (**c**) Human *VTN* (Vitronectin), and (**d**) Human *RUNX2* (Runt-related transcription factor 2) mRNA specific transcript levels evaluated with quantitative RT-PCR in h-osteoblasts cultured for 2 days. HT refers to non-functionalized Sr/Mg-doped HT foams, and SIL refers to silanized scaffolds; **** *p*-value < 0.0001.

Human *SPP1* (secreted phosphoprotein 1, Osteopontin), Human *VTN* (Vitronectin), Human *IBSP* (Integrin-Binding Sialoprotein), and Human *RUNX2* (Runt-related transcription factor 2) mRNA specific transcript levels were evaluated with quantitative RT-PCR at 2 days after h-osteoblast seeding. The results are reported in Figure 12.

Compared to the controls, Sr/Mg-doped HT foams functionalized with D2HVPF showed an increase in expression of *SPP1*, *VTN*, *RUNX2*, and *IBSP*, which are gene coding proteins fundamental for bone formation.

## 4. Discussion

Preliminary in vitro bioassays on Sr/Mg-doped hardystonite foams functionalized via side-chain protected peptide produced sub-optimal results, especially in terms of cell viability.

No proliferation was detected at days 2, 4, or 6 after h-osteoblasts seeding on all covalently and selectively functionalized HT foams via side-chain protected peptide D2HVP. Furthermore, comparing the images taken after 6 days from the seeding using the Operetta CLS high-content microscope, it was quite evident that non-functionalized HT samples were colonized by many more cells than the functionalized samples (Figure 6).

Moreover, h-osteoblasts seeded on simple Sr/Mg-doped HT foams appeared more spread out, whilst cells on functionalized foams showed a more rounded morphology, likely being on a harsher surface. Cells seeded on silanized HT foams showed the same elongated morphology as h-osteoblasts seeded on non-functionalized foams (Figure 6), suggesting that the change in cell behavior was probably due to the following functionalization steps.

One possible explanation for these results is that Sr/Mg-doped HT might be more sensitive to the acid treatment used to covalently graft D2HVP with respect to wollastonite/diopside [6], wherein the same functionalization protocol showed bioactivity improvement. Although the vibrational techniques revealed the effective anchoring of the peptide, Raman and IR findings confirmed the partial degradation of the bioceramic Mg/Sr-doped HT substrate when it was functionalized via side-chain protected peptide D2HVP.

In addition, ICP analysis was carried out to understand whether the functionalization treatment via side-chain protected peptide modified the composition of the bioceramic, inducing the release of ions into the cell medium, shifting the bioceramic’s ion balance toward a higher percentage of Zn that could be cytotoxic if de-adsorbed up to a 0.05 mg/mL concentration [52,53]. Based on the ICP results, the Sr/Mg-doped HT functionalized via side-chain protected D2HVP released all the ions of which it is made, except Si, at all experimental times. Focusing on Zinc release at all time points, the concentration was higher than the 0.05 mg/mL limit at all time points.

In bioglasses, partially replacing Sodium with Zinc was demonstrated to induce osteoblast proliferation, differentiation, and improve the bone-bonding ability [55]. It is well established that Zinc is a paramount element for cell development, growth, and differentiation [56], since it encourages protein synthesis, and it is of crucial importance in DNA replication. It also acts as a cofactor for several enzymes [57]. Zinc is also involved in the activation of bone formation [58] and in the inhibition of bone resorption [59]. Another important role played by Zinc, which promotes its incorporation in bioglasses, is its ability to prolong their chemical durabilities by slowing the glass dissolution and improving the glasses’ mechanical properties [60].

However, a study from Aina V. et al. demonstrated that the consequence of the presence of Zinc on glass durability is insufficient to avoid the complete dissolution of the material [52]. The scaffold dissolution causes the release of Zn^2+^ ions in solution, which might be relevant in eliciting changes in cell functions and metabolism when occurring in vivo. In fact, Aina et al. demonstrated that the presence of Zn enhances, in a dose-dependent manner, the LDH release in the extracellular medium (a cytotoxicity index), the accumulation of intracellular MDA (a lipoperoxidation index), and augments the HO-1 expression and PPP activity (signals of cell response to oxidative stress) [52]. All these considerations, combined with the results from vibrational spectra and the ICP analysis, support that the first functionalization strategy via side-chain protected peptide and particularly its acid treatment (TFA) induced a modification of the bioceramic structure, which in turn caused a cell toxic release of ions.

Regarding Sr, as a trace element (nearly 0.00044% of 70-kg standard man body mass corresponding to ~0.32 g [61]) in the human body, it has been shown to produce beneficial impacts on bone development, inducing osteoblast activity, by stimulating bone formation [62,63,64,65] and reducing bone resorption [66,67]. The amount of Stronzium in the skeleton is only 0.035 of its Ca content [61]. By now, the overdosing of Sr has not been related to toxic symptoms in men. Nevertheless, the intravenous administration of Stronzium at high doses can induce hypocalcemia [61,68]. The influence of high doses of Stronzium in bone was further confirmed by Verberckmoes et al. in an in vitro study [67]. The latter showed a defective formation of hydroxyapatite in the presence of high Sr concentration, which creates special osteomalacia problems. The use of Sr ions as a sintering additive to improve PbTiO_3_ ceramic density has also been reported [69], decreasing the dissolution rate of the ceramics [70]. It was demonstrated that Sr-doped CaSiO_3_ ceramics possess improved physical and biological properties compared to pure CaSiO_3_ ceramics [71].

Magnesium ions have been demonstrated to stimulate new bone formation [72] and increase bone cell adhesion and stability (probably due to interactions with integrins) [12,72,73]. Bioactive glasses doped with Mg have been shown to improve dissolution behavior due to the Mg effect disrupting the silica network and exhibiting decreased crystallization of the HAp layer on the bioglass surface [74].

To overcome non-encouraging results and to indirectly correlate the results to using acid solutions during D2HVP grafting, we designed a different covalent and selective functionalization strategy that, after sample silanization, required only mild reaction conditions and aqueous solutions, avoiding any acid treatment. Vibrational Raman and IR spectroscopies confirmed the presence of the anchored peptide on the bioceramic surface.

This strategy involved a simple modification of the D2HVP peptide’s sequence (D2HVPF), introducing an aldehyde group at the C-terminus, which can selectively react with the amine groups of the bioceramic silanized surfaces. ICP analysis confirmed that Sr/Mg-doped HT samples functionalized via aldehyde peptide showed no ion release over time. Furthermore, foams functionalized with this second strategy significantly increased h-osteoblast proliferation at 6 days. DHVPF-grafted scaffolds enhanced gene expression of *SPP1*, *VTN*, *IBSP*, and *RUNX2* more than 10 times compared to silanized and pristine HT at 2 days post-seeding. Finally, LDH assay confirmed the absence of cytotoxicity at 6 days post h-osteoblast seeding, thus indicating that the strategy via aldehyde terminus is a simple and effective chemical procedure for the covalent and selective grafting of a biomolecule onto a bioceramic surface to improve its bioactivity.

## 5. Conclusions

In this work, the protocol for selective functionalization of Sr/Mg-doped HA with a protease-resistant adhesive peptide was successfully optimized. The failure of a previously satisfactory protocol on Wollastonite/Diopside bioceramic foams was explained by the massive release of ions, especially Zinc, whose concentrations exceeded the threshold value for cell cytotoxicity.

The alternative functionalization strategy proposed required a simple modification of the adhesive peptide sequence that introduced an aldehyde group at the peptide C-terminus. This single modification allows (i) avoidance of organic solutions and the final acid treatment necessary to remove side-chain protecting groups of the peptide and (ii) introduction of a selective group (aldehyde group) directly as part of the peptide backbone, reducing the full functionalization procedure to one single reaction. Functionalized HT obtained with this method showed increased h-osteoblast proliferation (6 days) and gene expression (2 days) when compared to the controls. No ion release was detected for Sr/Mg-doped HT functionalized via aldehyde peptide, and the LDH assay confirmed the absence of cytotoxicity at 6 days post h-osteoblast seeding. In conclusion, the novel method via aldehyde peptide represents a valuable strategy for easily anchoring bioactive molecules to acid-sensitive substrates.

## Figures and Tables

**Table 1:** Oligonucleotides used in qPCR experiments. ^a^ Fw: forward; ^b^ Rv: reverse.

GENE [ACCESSION#]	SEQUENCE
GAPDH	^a^ Fw: 5′-cgggaagcccatcacca-3′
[NM_002046]	^b^ Rv: 5′-ccggcctcaccccatt-3′
IBSP	Fw: 5′-ttactaccaccaccagtgaagc-3′
[NM_004967]	Rv: 5′-gatgcaaagccagaatggat-3′
VTN	Fw: 5′-ggaggacatcttcgagcttct-3′
[NM_000638]	Rv: 5′-gctaatgaactggggctgtc-3′
SPP1	Fw: 5′-aagtttcgcagacctgacatc-3′
[NM_000582]	Rv: 5′-ggctgtcccaatcagaagg-3′
RUNX2	Fw: 5′-cagtgacaccatgtcagcaa-3′
[NM_001024630]	Rv: 5′-gctcacgtcgctcattttg-3′

## Data Availability

Not applicable.

## Supplementary Materials

The following supporting information can be downloaded at https://www.mdpi.com/article/10.3390/biomimetics8020185/s1: Figure S1: Raman spectra of the Sr/Mg-doped HT foams via side-chain protected peptide; Figure S2: IR spectra of the Sr/Mg-doped HT foams via side-chain protected peptide; Figure S3: SEM-EDS analysis of silanized Sr/Mg-doped HT; Figure S4: SEM-EDS analysis of Sr/Mg-doped HT functionalized via protected peptide D2HVP; and Figure S5: SEM-EDS analysis of Sr/Mg-doped HT functionalized via aldehyde peptide D2HVPF.
