# Supplementary material for: Proteolytically Resistant Bioactive Peptide-Grafted Sr/Mg-Doped Hardystonite Foams: Comparison of Two Covalent Functionalization Strategies
Source: Biomimetics (Basel). 2023 Apr 29;8(2):185. doi: 10.3390/biomimetics8020185 (PMC10204510; doi:10.3390/biomimetics8020185)
Supplement: Supplementary file 1 [file biomimetics-08-00185-s001.zip › biomimetics-2274363-supplementary.pdf]

biomimetics

MDPI

Article

Proteolytically Resistant Bioactive Peptide-Grafted Sr/Mg-Doped Hardystonite Foams: Comparison of Two Covalent Functionalization Strategies

Annj Zamuner [1,2,*], Elena Zeni [2], Hamada Elsayed [2], Michele Di Foggia [3], Paola Taddei [3], Antonella Pasquato [2], Lucy Di Silvio [4], Enrico Bernardo [2], Paola Brun [5] and Monica Dettin [2]

[1] Department of Civil, Environmental, and Architectural Engineering, University of Padua, Via Marzolo 9, 35131 Padova, Italy
[2] Department of Industrial Engineering, University of Padua, Via Marzolo 9, 35131 Padova, Italy; monica.dettin@unipd.it (M.D.)
[3] Department of Biomedical and Neuromotor Sciences, University of Bologna, Via Irnerio 48, 40126 Bologna, Italy
[4] Faculty of Dentistry, Oral & Craniofacial Sciences King's College London, London SE1 9RT, UK
[5] Department of Molecular Medicine, University of Padua, Via Gabelli 63, 35121 Padova, Italy
* Correspondence: annj.zamuner@unipd.it; Tel.: +39-049-8275553

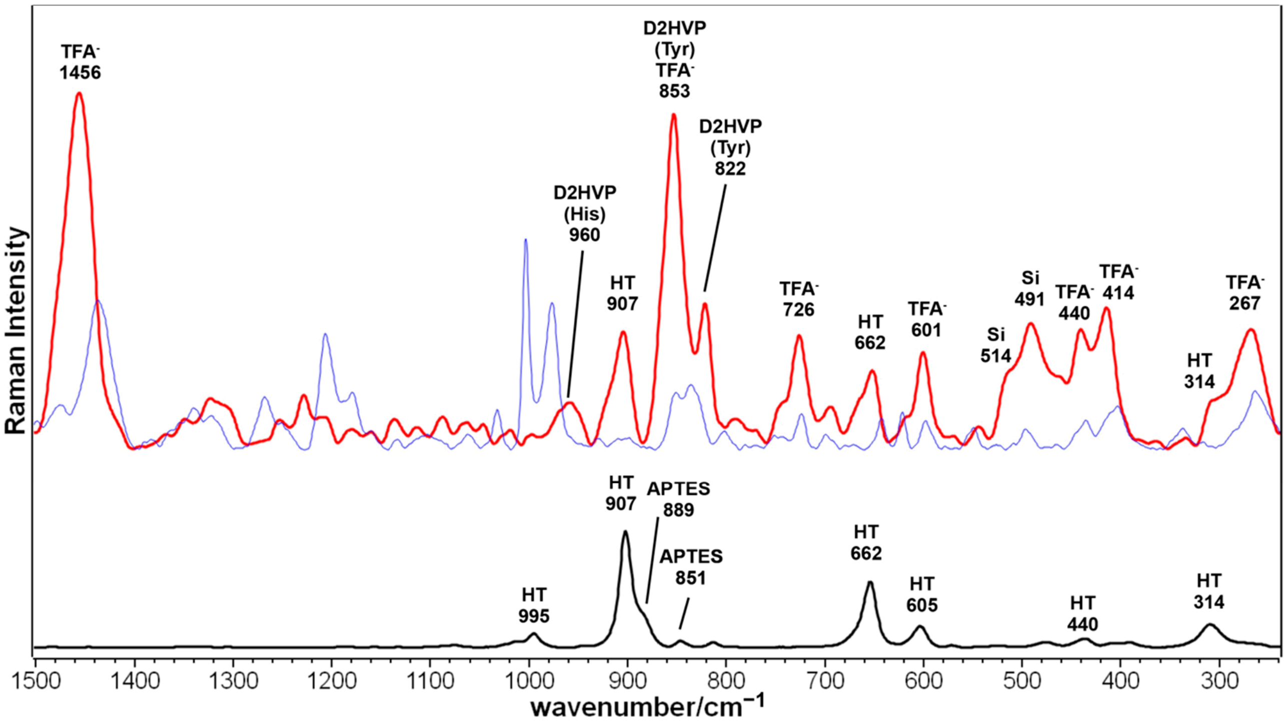

Figure S1. Raman spectra of the Sr/Mg-doped HT foams as obtained after silanization treatment (black line) and after the covalent grafting via side-chain protected peptide D2HVP (red line). The spectrum of the pure D2HVP peptide is reported for comparison (blue line). The bands assignable to the bioceramic substrate (HT), to amino acid residues belonging to the D2HVP peptide (tyrosine (Tyr) and histidine (His)), trifluoroacetate anion (TFA^-) and silica (Si) are indicated.

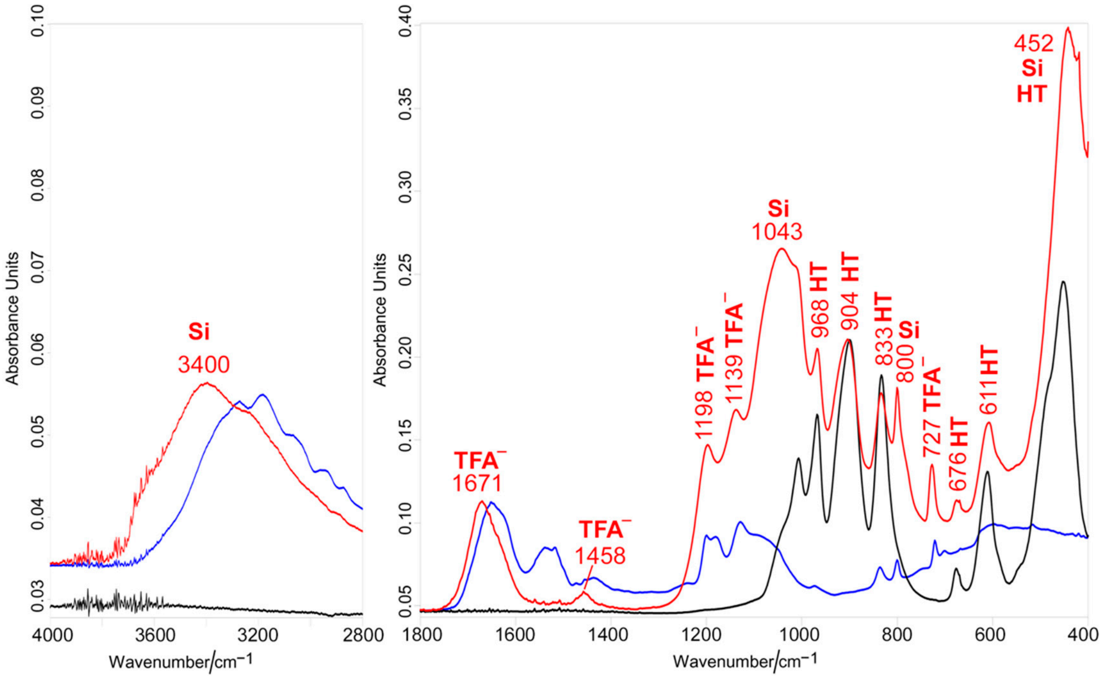

Figure S2. IR spectra of the Sr/Mg-doped HT foams as obtained after silanization treatment (black line) and after the covalent grafting via side-chain protected peptide D2HVP (red line). The spectrum of the pure D2HVP peptide is reported for comparison (blue line). The bands assignable to the bioceramic substrate (HT), trifluoroacetate anion (TFA^-) and silica (Si) are indicated.

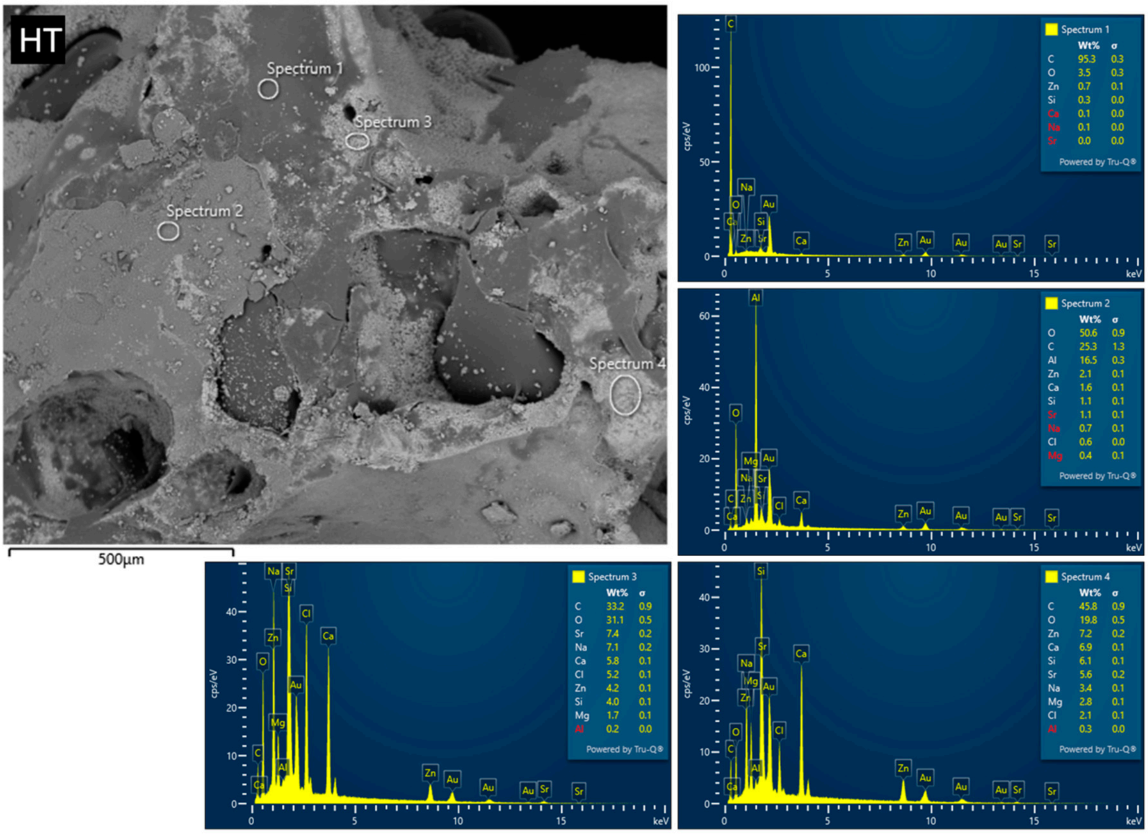

Figure S3. SEM-EDS analysis of silanized Sr/Mg-doped HT, after 4 months in 20 mM $NaH_2PO_4 \cdot H_2O$ and 200 mM NaCl buffer.

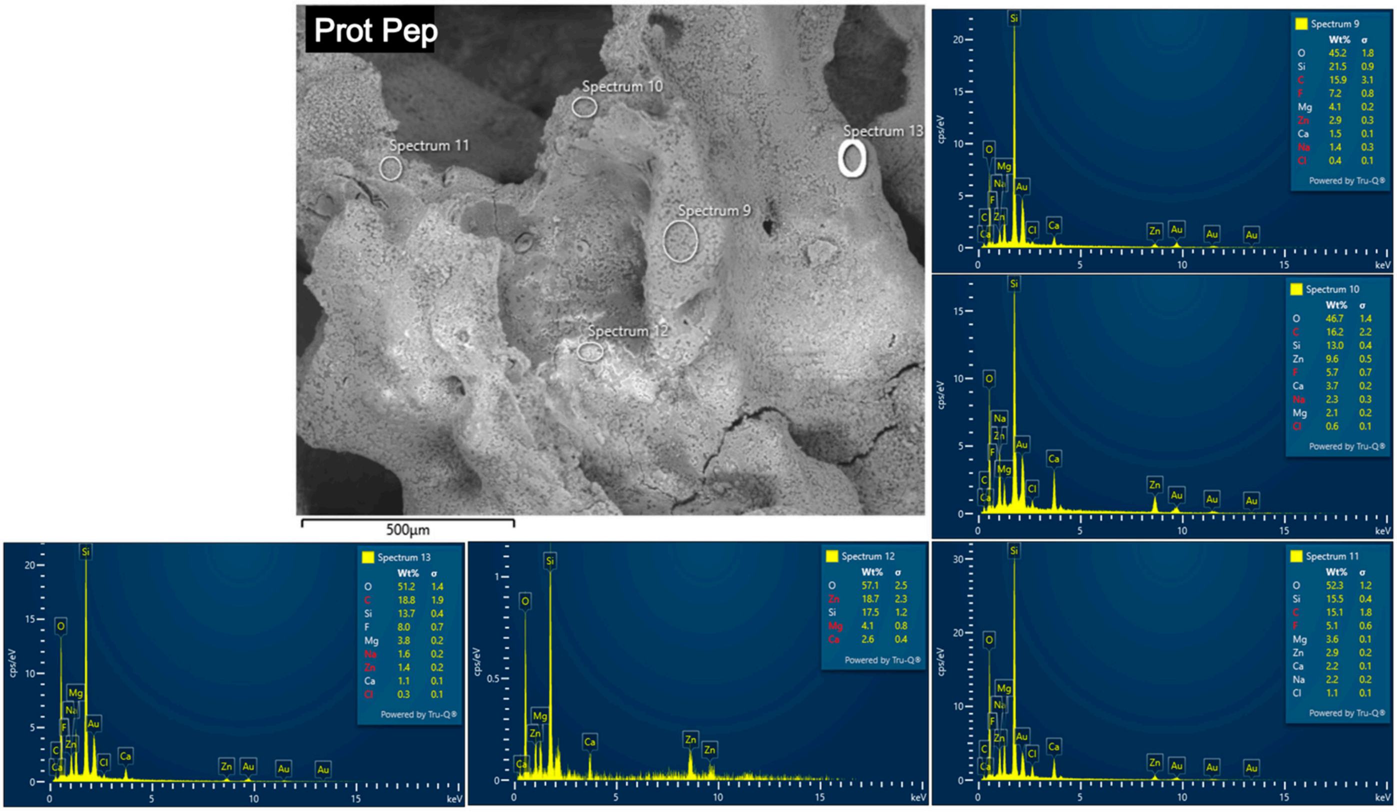

Figure S4. SEM-EDS analysis of Sr/Mg-doped HT functionalized via protected peptide D2HVP, after 4 months in 20 mM $NaH_2PO_4 \cdot H_2O$ and 200 mM NaCl buffer.

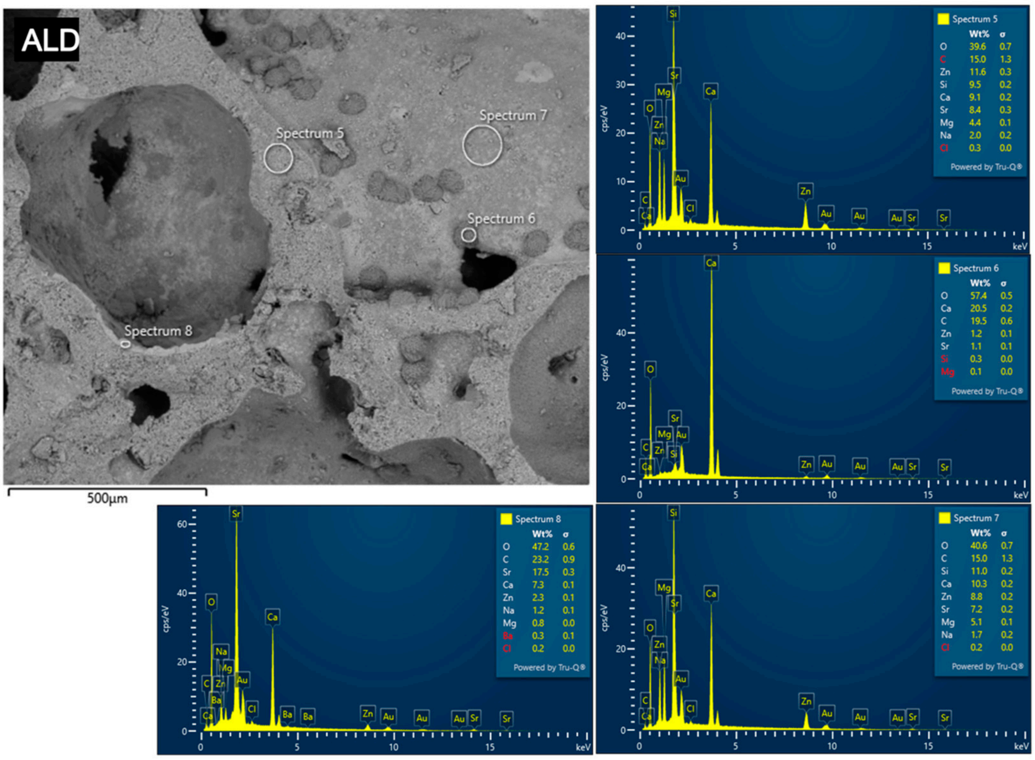

Figure S5. SEM-EDS analysis of Sr/Mg-doped HT functionalized via aldehyde peptide D2HVPF, after 4 months in 20 mM $NaH_2PO_4 \cdot H_2O$ and 200 mM NaCl buffer.
